# High-fat diet causes mechanical allodynia in the absence of injury or diabetic pathology

**DOI:** 10.1038/s41598-022-18281-x

**Published:** 2022-09-01

**Authors:** Jessica A. Tierney, Calvin D. Uong, Melissa E. Lenert, Marisa Williams, Michael D. Burton

**Affiliations:** Laboratory of Neuroimmunology and Behavior, Department of Neuroscience, School of Behavioral and Brain Sciences, Center for Advanced Pain Studies (CAPS), University of Texas at Dallas, 800 W. Campbell Road, Richardson, TX 75080 USA

**Keywords:** Neuroimmunology, Feeding behaviour, Immunology, Neuroscience

## Abstract

Understanding the interactions between diet, obesity, and diabetes is important to tease out mechanisms in painful pathology. Western diet is rich in fats, producing high amounts of circulating bioactive metabolites. However, no research has assessed how a high-fat diet (HFD) alone may sensitize an individual to non-painful stimuli in the absence of obesity or diabetic pathology. To investigate this, we tested the ability of a HFD to stimulate diet-induced hyperalgesic priming, or diet sensitization in male and female mice. Our results revealed that 8 weeks of HFD did not alter baseline pain sensitivity, but both male and female HFD-fed animals exhibited robust mechanical allodynia when exposed to a subthreshold dose of intraplantar Prostaglandin E_2_ (PGE_2_) compared to mice on chow diet. Furthermore, calcium imaging in isolated primary sensory neurons of both sexes revealed HFD induced an increased percentage of capsaicin-responsive neurons compared to their chow counterparts. Immunohistochemistry (IHC) showed a HFD-induced upregulation of ATF3, a neuronal marker of injury, in lumbar dorsal root ganglia (DRG). This suggests that a HFD induces allodynia in the absence of a pre-existing condition or injury via dietary components. With this new understanding of how a HFD can contribute to the onset of pain, we can understand the dissociation behind the comorbidities associated with obesity and diabetes to develop pharmacological interventions to treat them more efficiently.

## Introduction

As medicine shifts its focus towards sustainable lifestyle changes to combat disease, diet is often the first criteria to be evaluated since its composition influences the health status of an individual. For example, a Western diet consisting primarily of saturated fats that contribute to the increasing levels of obesity, diabetes, and comorbidities in Western countries^1–3^. Individuals who consume high amounts of saturated fats possess high amounts of broken-down circulating free fatty acids (FFAs) in their bloodstream. Recently, it has been shown that a HFD increases existing mechanical pain hypersensitivity independent of obesity^4,5^. However, it is still unclear if a HFD has the pain plasticity altering machinery that can sensitize individuals to respond to subthreshold stimuli and what it could mean for clinical populations earlier in diagnosis.

Individuals consuming a Western diet over time manifest a variety of symptoms and morbidities, including metainflammation, obesity, and diabetes^1,6^. Exposure to saturated fatty acids found in a Western diet induces systemic inflammation via pro-inflammatory cytokine release such as tumor necrosis factor-α (TNF-α) and interleukin-6 (IL-6)^7–13^. Several studies demonstrate how HFD exposure can exacerbate pre-existing conditions, such as post-operative pain and inflammatory-induced pain and can hinder recovery from injury, indicating that components of the HFD may have a direct role in pain development^5,14–17^. However, no studies have investigated how HFD alone can be sensitizing factor to induce robust pain states to non-painful stimuli. Understanding how diet contributes to the development of neuronal sensitivity will alter traditional medical approaches and will also aid in the prevention of chronic neuropathy in patients with diabetes. However, the transition towards chronic painful neuropathy in patients with severe diabetic pathology and the role in which diet plays into this is not well understood. In order to understand the complex dynamics of how diet can be a sensitizing factor and play a role in diabetic pathology, we employed a modified hyperalgesic priming model. The model is where an injury or stimulus sensitizes individuals to a secondary innocuous stimulus after full recovery (Aley et al., 2000; Reichling and Levine, 2009). The model studies the mechanisms involved in the transition from acute to chronic pain states and illustrates how an otherwise healthy individual can become sensitized to an innocuous stimulus, typically a subthreshold dose of PGE_2_^18,25^. However, no studies have investigated whether HFD alone can be used as the initial insult to prime an individual even in the absence of a prior injury or pre-existing conditions, which differs from the typical hyperalgesic priming model since the initial insult, the short exposure to the HFD alone does not induce mechanical hypersensitivity. In previous studies, following the initial stimulus, neuronal injury and sensitization drives a hyperalgesic primed state to trigger neuroplastic changes in isolectin B4-positive (IB4+) nociceptors^18–25^. We hypothesized that short-term consumption of a HFD prior to the development of chronic hyperglycemia or obesity—acts as an initial non-painful insult to induce subsequent painful hyperalgesic priming. This result would be paradigm shifting as sensitization is typically accompanied by symptomology and would dictate pathology before symptomology in this case.

## Results

### Short-term HFD does not cause obesity or hyperglycemia

**Figure 1:**
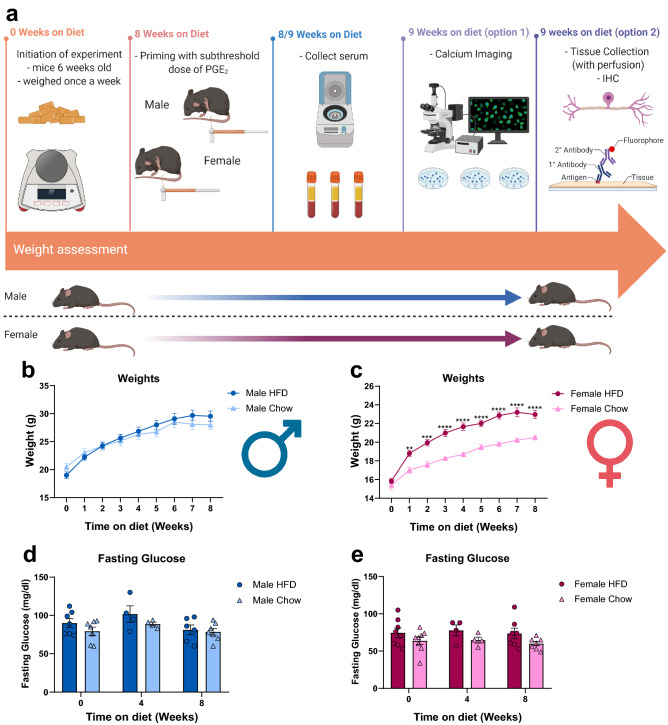
A short term HFD regime does not induce the development of obesity nor hyperglycemia. (**a**) Mice were assigned either a HFD or chow diet at 6 weeks of age for 9 weeks. Weight assessment was performed weekly and blood glucose measurements were performed every weeks starting at week zero. On week 8, mice received a subthreshold dose (0.005 mg/mL) of PGE_2_ via intraplantar injection and mechanical hypersensitivity was measured afterwards using von Frey. In addition, serum was collected on week 8 and 9 to assess plasma FFA levels. On week 9, tissues were collected and were used either for calcium imaging or immunohistochemistry. (**b**, **c**) HFD males (n = 14) did not gain significantly more weight than their chow counterparts (n = 14) whereas HFD females (n = 15) did gain significantly more weight than their chow counterparts (n = 14); both did not reach levels of obesity. *p < 0.05; **p < 0.01; ***p < 0.001; ****p < 0.0001 (Two-way ANOVA with Sidak’s post hoc comparison). (**d**, **e**) No significant developments occurred in their fasting glucose levels between HFD males (n = 7) and chow males (n = 7) nor between HFD females (n = 8) and chow females (n = 8).

Mice were randomly assigned either a HFD or chow diet at 6-weeks and were weighed weekly to confirm that mice are not developing an obese phenotype during this HFD regime (Fig. 1). No overt biological differences (obesity) were observed (defined as gaining 20–30% more than their chow counterparts)^26^. HFD males had comparable weights to chow counterparts throughout the experiment (Fig. 1b), while HFD females did show modest weight gain (F(1, 27) = 33.91, p < 0.0001) (Supplementary Table 1), but not to “obese” measures (~ 8%/week changes) (Fig. 1c)^27^. Moreover, none of the females reached > 25 g, which for C57/B6 females in this age range are considered obese^27,28^.

Mice from both diets were then tested to measure glycemia levels. Fasting glucose levels were assessed after a 14 h fast at the beginning of the light cycle, at 4 and 8 weeks on diet (Fig. 1). At all timepoints, there was no significant effect of HFD on fasting glucose levels when compared to their chow sex-matched counterpart. These results confirm previous findings that a short-term exposure to a HFD does not induce the development of obesity or hyperglycemia.

### Short-term HFD causes PGE_2_-induced sensitization

**Figure 2:**
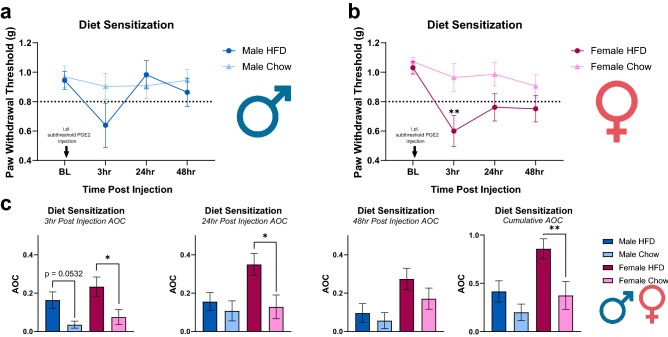
A short-term HFD regime causes diet sensitization. Mice received intraplantar injections of subthreshold dose of PGE_2_ on week 8. (**a**, **b**) HFD males (n = 10) and females (n = 11) showed a decrease in paw withdrawal threshold post subthreshold PGE_2_ injection compared to chow males (n = 11) and females (n = 10), respectively. **p < 0.01 (Two-Way ANOVA with Sidak’s post hoc comparison (**c**) area over the curve (AOC) for each time point showed that the three-hour time point elicited the largest response to a subthreshold dose of PGE_2,_ specifically in females. *p < 0.05 (Two-way ANOVA with Sidak’s post hoc comparison). Total AOC showed females significantly sensitized to the HFD compared to chow females. **p < 0.01 (Two-way ANOVA with Sidak’s post hoc comparison).

After 8 weeks on diet, behavioral measurements were taken, and HFD animal behavior was observed to be no different than their chow counterparts. All animals were then injected (intraplantar) with a subthreshold dose of PGE_2_. Administration of a subthreshold dose of PGE_2_ caused a decrease in paw withdrawal threshold in HFD males and females and was analyzed with consideration towards the influence of diet, sex, and time and the interactions between each factor (Fig. 2). There was a significant effect of time (p = 0.0039) and diet (p = 0.0024) on paw withdrawal threshold but no significant interactions were observed between sex, sex and diet, and time and diet. Analyzing the area over the curve (AOC) at each time point showed that 3-h post PGE_2_ injection elicited a significant effect of diet (F (1, 38) = 13.22), p = 0.0008) with significant difference revealed between HFD and chow females (p = 0.0142) while HFD males reported robust differences from chow males (p = 0.0535) (Supplementary Table 2). The 24-h post PGE_2_ injection observed a significant effect of diet (F(1, 38)) = 6.022 with significance difference between HFD and chow females (p = 0.0141). Total AOC analysis revealed a significant effect of diet (F (1, 38) = 9.939, p = 0.0032) with a significant difference shown between HFD and chow females (p = 0.0077) while no significant difference is shown between HFD and chow males (p = 0.3210) (Supplementary Table 2). Taken together, a short-term HFD alone does not induce a painful phenotype but does sensitize mice to innocuous stimuli (allodynia) in the absence of pre-existing pain conditions, obesity, or diabetic pathology.

### Short-term HFD sensitizes DRG sensory neurons to become more responsive to capsaicin

**Figure 3:**
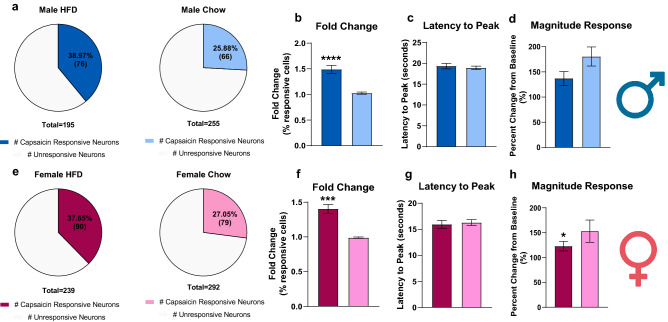
HFD fed males and females have an increased response rate to capsaicin after a short-term HFD regime. (**a**, **e**) HFD males (n = 6 mice, 195 cells) and females (n = 8 mice, 239 cells) had a significant response (data not shown) rate to capsaicin (males: 38.97% 76 capsaicin-responsive neurons/195 neurons; females: 37.65%, 90 capsaicin-responsive neurons/239 neurons) in comparison to their chow counterparts (males: 25.88%, 66 capsaicin-responsive neurons/255 neurons; females: 27.05%, 79 capsaicin-responsive neurons/292 neurons) (Chow males: n = 9 mice, 255 cells; Chow females: n = 7 mice, 292 cells). (**b**, **f**) HFD males and females had a significant difference in fold change compared to their chow counterparts. ***p < 0.001; ****p < 0.0001 (Two-way ANOVA with Sidak’s post hoc comparison). (**c**, **g**) There was no significant diet-dependent effect on the latency to maximum capsaicin response, but (**d**, **h**) there was a significant diet-dependent effect on response magnitude. *p < 0.05 (Two-way ANOVA with Sidak’s post hoc comparison).

DRGs were collected and cultured for calcium imaging experiments to measure their response to capsaicin (Fig. 3). Roughly, 25–30% of DRG neurons are TRPV1 positive which means they respond to capsaicin, but this response rate increases when neurons become sensitized after injury or pre-existing condition^29–33^. Cultured DRG sensory neurons from both HFD males and females had a significant increase in their response rate to capsaicin in comparison to their chow counterparts; males HFD (38.97%, 76 capsaicin-responsive neurons/195 neurons) vs. chow (25.88%, 66 capsaicin-responsive neurons/255 neurons) (p = 0.0041) (Fig. 3a), and females HFD (37.65%, 90 capsaicin-responsive neurons/239 neurons) vs. chows (27.05%, 79 capsaicin-responsive neurons/292 neurons) (p = 0.0114) (Fig. 3e). Additionally, there was a significant diet effect that showed an increase in the fold change (F (1,16) = 71.58, p < 0.0001) in HFD males (p < 0.0001) (Fig. 3b) and in HFD females (p < 0.0001) (Fig. 3f) and a significant diet effect that showed a decrease in magnitude (peak) response (F (1, 306) = 8.166, p = 0.0046) with significant differences revealed between HFD and chow females (p = 0.0434) (Fig. 3h) (Supplementary Table 3). This reveals that short-term HFD exposure enhances DRG sensory neuron’s sensitivity to capsaicin, resulting in an increased response rate, number of cells activated, and strength of the activation.

### Short-term HFD induces an increase in the neuronal marker of injury, ATF3, in DRG neurons

**Figure 4:**
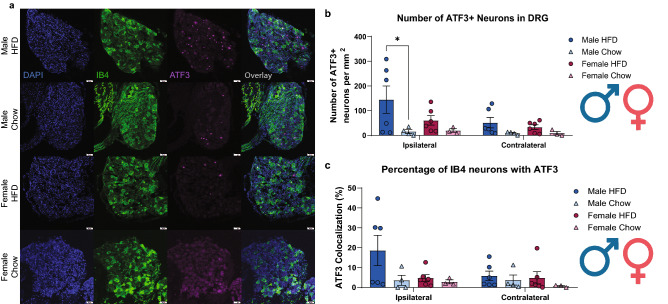
A short-term HFD regime induces ATF3 upregulation in lumbar DRGs. (**a**) Ipsilateral lumbar (L3-L5) DRGs from both diet groups were immunostained with DAPI (blue), IB4+ (green), and ATF-3 (purple). (**b**) There was a diet-dependent increase in ATF3 expression in HFD males (n = 6) and females (n = 6) compared to their chow male (n = 4) and female (n = 3) counterparts in both ipsilateral and contralateral lumbar DRG; however, only the HFD males had a significant ATF3 upregulation compared to their chow males in the ipsilateral DRG. There was no significant difference in ATF3 expression between ipsilateral and contralateral DRG. *p < 0.05 (Three-way ANOVA with Sidak’s post hoc multiple comparison). (**c**) There was an observed increase in ATF3 expression in IB4+ nociceptors in HFD males and females compared to their chow counterparts, but it did not reach the threshold of significance. Scale bar: 50 μm.

At the end of the study, lumbar (L3-L5) DRGs were collected (they innervate the hind paw) and were used for IHC experiments to assess for neuronal injury, using ATF3 in DRG neurons (Fig. 4). ATF3 is low to no expressing in chow tissue but is upregulated in HFD DRGs. The number of ATF3-expressing neurons was recorded and normalized by area of the DRG and was analyzed with consideration towards the influence of diet, sex, and PGE_2_ injection and the interactions between each factor. There was a significant effect of diet on ATF3 expression in the ipsilateral DRG (F (1, 30) = 7.620, p = 0.0098) but comparisons to the contralateral DRG revealed no significant interaction between diet and PGE_2_ (Supplementary Table 4). Moreover, there was significant ATF3 upregulation in HFD males when compared to chow males (p = 0.0421) but no robust differences between HFD and chow females were measured (Supplementary Table 4). Interestingly, assessment of IB4+ nociceptors expressing ATF3 after the short-term HFD regime revealed an almost significant effect of diet on the number of IB4 + nociceptors expressing ATF3 (F (1, 30) = 3.588, p = 0.0679) (Supplementary Table 4). This reveals that a short-term HFD exposure can cause neuronal injury in the DRG, especially in IB4+ nociceptors.

### Short-term HFD does not induce macrophage or satellite glial cell activation in the DRG

**Figure 5:**
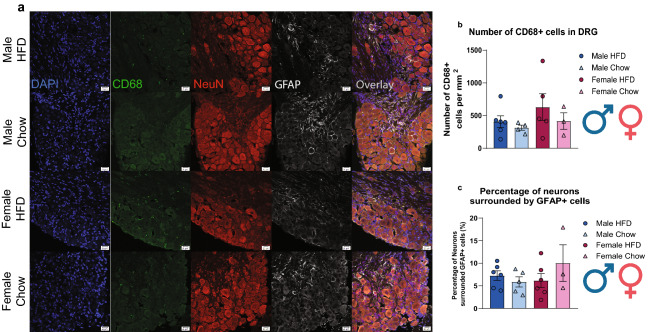
Diet sensitization does not alter monocyte infiltration nor satellite glial cell activation in the lumbar DRGs. (**a**) Ipsilateral lumbar (L3-L5) DRGs from both diet groups were immunostained with DAPI (blue), CD68 (green), NeuN (red), and GFAP (white). (**b**) There were no changes in monocyte infiltration nor (**c**) were there an increase in GFAP expression in satellite glial cells between HFD males (n = 6) and females (n = 5) and chow males (n = 4) and females (n = 3). Scale bar: 20 μm.

Further IHC experiments were performed to assess the level of macrophage activation/monocyte infiltration and satellite glial cell activation in the lumbar (L3-L5) DRGs (Fig. 5). CD68 was used to assess monocyte/macrophage infiltration into the DRG after a short-term HFD regime by measuring the number of CD68+ cells and normalizing it by the area of the DRG. There was neither a significant effect of diet nor a significant interaction between diet and subthreshold PGE_2_ in the number of macrophages in the DRG (Supplementary Table 5). Glial fibrillary acidic protein (GFAP), a marker for satellite glial cell activation, was used to assess the number of activated satellite glial cells after short-term HFD exposure by measuring the number of GFAP+ cells and normalizing it by the total number of neurons. Similarly, no significant effect of diet nor significant interaction between diet and subthreshold PGE_2_ in the number of activated satellite glial cells in the DRG (Supplementary Table 5). Collectively, this suggests that a short-term exposure to a HFD does not alter the number of macrophages in the DRG nor does it induce the activation of satellite glial cells.

### Short-term HFD led to an increase in saturated fatty acids which may precipitate the development of diet-induced sensitization

**Figure 6:**
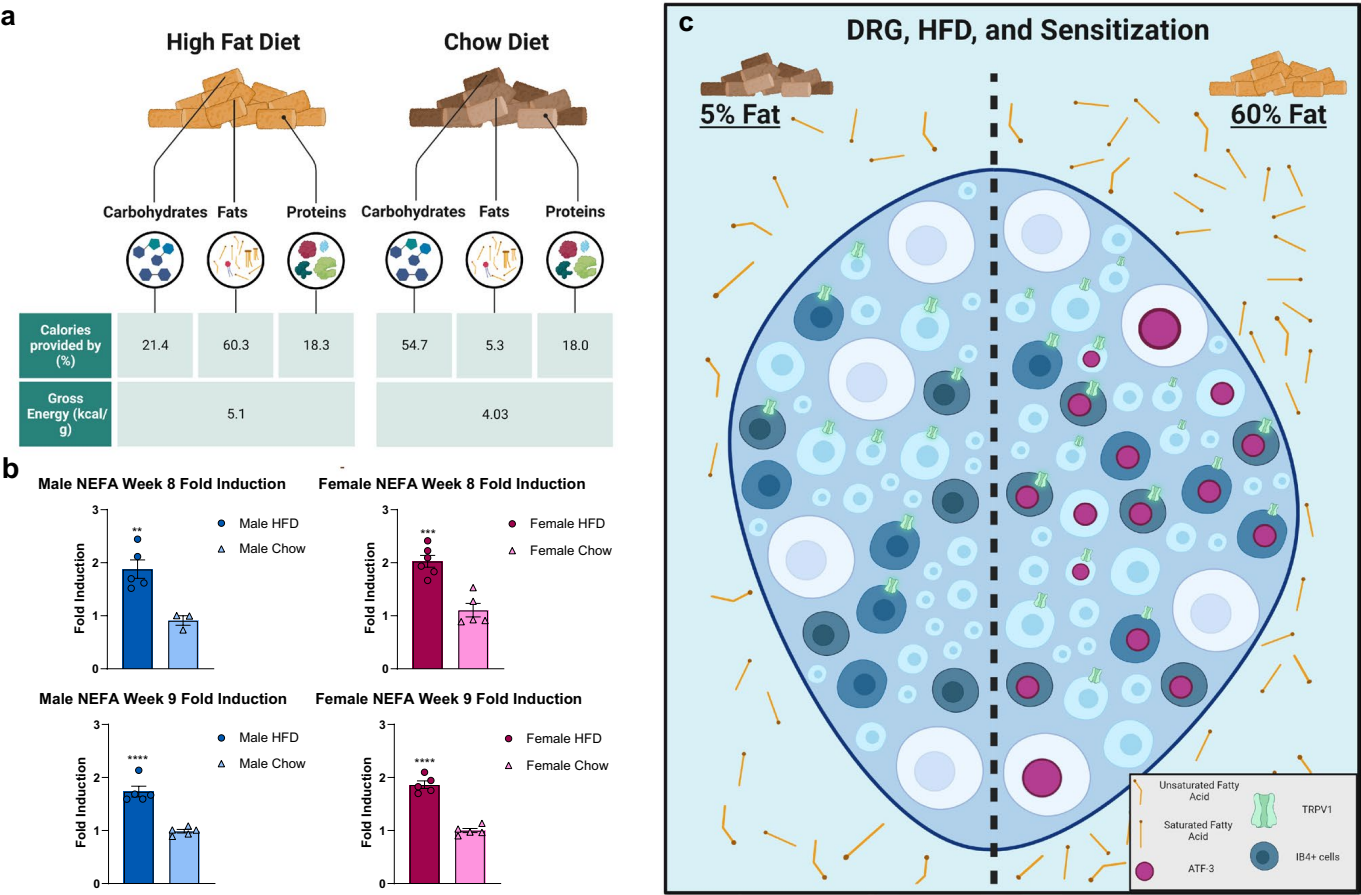
A short-term HFD regime led to an increase in saturated fatty acids which may precipitate the development of diet sensitization. (**a**) HFD males and (**b**) females had significantly higher levels of free fatty acids in their serum in comparison to their chow counterparts on week 8 and 9. *p < 0.05; **p < 0.01; ***p < 0.001; ****p < 0.0001 (two-way ANOVA with Sidak’s post hoc comparison). (**c**) An 8-week HFD regime was shown to be sufficient in sensitizing mice to the subthreshold dose of PGE_2_ irrespective of sex. A direct consequence of a short-term HFD regime led to an increase in percentage of capsaicin-response neurons and ATF3 upregulation in DRG neurons, especially in IB4+ nociceptors. Mice on the short-term HFD regime had an observed increase in serum FFA levels from the short-term HFD regime, which primarily consists of saturated fatty acids.

Serum was collected from mice on week 8 and 9 on a HFD or chow diet to assess plasma FFA levels (Fig. 6). A majority of the calories provided by the HFD is derived from fats, especially saturated fatty acids, with 60.3% of its calories deriving from this macronutrient. In comparison, the chow diet derives 5.3% of its calories from fats with the majority derived from carbohydrates (54.7%). The difference in the macronutrient composition between the 2 diets motivated a follow-up experiment to measure the levels of plasma FFA between the diet groups. A significant effect of diet on plasma FFA levels was observed on week 8 (F (1, 15) = 44.13, p < 0.0001) and 9 (F (1, 16) = 143.2, p < 0.0001). HFD males had a significant fold induction increase for non-esterified fatty acid (NEFA) in comparison to the chow males on week 8 (HFD males: 1.88 Fold Induction, 94.61 μM NEFA; Chow males: 0.91 Fold Induction, 51.97 μM NEFA) (p = 0.0010) and 9 (HFD Males: 1.74 Fold Induction, 104.34 μM NEFA; Chow Males: 0.99 Fold Induction, 62.51 μM NEFA) (p < 0.0001) (Supplementary Table 6). Similarly, there was a robust fold induction increase for NEFA between HFD females and chow females on week 8 (HFD Females: 2.03 Fold Induction, 92.45 μM NEFA; Chow Females: 1.11, 44.73 μM NEFA) (p = 0.0003) and 9 (HFD Females: 1.86 Fold Induction, 103.97 μM NEFA; Chow Females: 1 Fold Induction, 49.14 μM NEFA) (p < 0.0001) (Supplementary Table 6). The augmented plasma FFA levels after a short exposure to a HFD may be inducing neuronal injury in the DRGs, increasing the number of capsaicin-responsive neurons, and precipitating the development of mechanical allodynia.

## Discussion

The aim of the study is to investigate if HFD alone can be a sensitization factor that drives pain or allodynia in the absence of obesity or diabetic pathology. Using diet as our priming catalyst, we demonstrated that a normally non-noxious stimuli can induce allodynia in the absence of a prior painful priming event. Typically, in hyperalgesic priming models the initial insult causes an overtly painful state that is fully recovered before a non-noxious stimulus is administered to elicit the “primed” state^25,34^. Our results revealed that consuming a diet rich in saturated fats for 8 weeks—prior to the development of obesity or diabetes—is sufficient to induce mechanical allodynia to a subthreshold dose of PGE_2_ in both males and females (Fig. 6).

Previous studies have demonstrated that consuming a HFD diet can exacerbate pre-existing pain states and prolong recovery from injury, such as plantar incision or an inflammatory insult ^5,14,17^. The Totsch et al., study referenced illustrates that an inflammatory stimulus can cause mechanical hypersensitivity from an inflammatory insult after 13 weeks on HFD. This model at 13 weeks would be considered a long-term HFD compared to our short-term paradigm. The study details how a HFD can cause obesity and worsen the pain from an inflammatory insult and shows mechanical hypersensitivity in both the HFD and chow groups, unlike our study. The Song et al., studies referenced have a similar timeline to the experiment conducted in this study. They show that a HFD can induce mechanical hypersensitivity from an inflammatory insult or post-surgical model in the absence or presence of obesity respectively. However, all of these models cause mechanical hypersensitivity in the HFD animals and the chow animals. The objective of this study was to show that in the place of the typical initial pain inducing insult in the hyperalgesic priming model that an 8 week course of HFD could cause allodynia when a subthreshold dose of PGE2 was administered. Importantly, our model does not induce pain in the chow groups, only in the HFD groups. A short-term paradigm of HFD in the absence of obesity or hyperglycemia was able to precipitate pain not just worsen it. These findings highlight how an individual consuming a Western diet does not have to display symptomology associated with obesity and/or diabetes to be at-risk of developing a more robust response to an already painful insult. However, no studies have investigated how HFD alone can be a sensitizing influence to induce robust pain states to non-painful stimuli. This distinction is important because it highlights the importance of understanding components of the Western diet and their effects on the body even before the development of any gross conditions. It is well-documented that saturated fatty acids (SFA) which are a large component of the Western diet, can directly activate immune cells and cause a robust pro-inflammatory cytokine production^1,35^. Yet investigations have only focused on the effects that this inflammatory diet can have when deleterious conditions like chronic inflammation, nerve damage, heart disease, etc. have already developed^3,6,8^. Our study seeks to rectify this by investigating how a HFD can induce allodynia, or primed state.

To be considered a “Western diet”, the diet must consists of at least 34% kcal from fat^36^, our diet consisted of 60% kcal from fat, with a formulation to reach prototypic levels of SFAs from Western diet. To investigate the role of SFAs, male and female WT C57BL/6J mice were randomly assigned either a HFD or chow diet at 6 weeks old and placed on these diets for 8 weeks. Weights and fasting glucose levels verified that none of the animals on HFD developed obesity or diabetic pathology during the short-term HFD experiment which corroborates previous findings^27,37^. Though HFD females did show a significant difference in weight from their chow counterparts, this does not equate to physiological obesity. Diet-induced obesity in females is defined as a weight > 25 g and females with no obese phenotype as < 23 g which none of the HFD females reached during the experiment (22.967 g ± 0.450 g)^27,28^. Previous studies have observed significant weight gain by week eight for WT C57BL/6J mice; however, there are several differences that makes our study different from previous literature. Since all mice used in this study were bred in-house and were placed in the same controlled housing conditions, there is minimal influence on whole-body metabolism as a result of the housing environment. The source and percentage of fats in our HFD regimen greatly differs from previous HFD studies, where numerous studies revealed that the source and percentage of fats in a HFD regimen can influence the metabolic outcome. In addition, correlational analysis between weights at week 8 on diet and mechanical allodynia revealed no significant correlation between the 2 variables in either sex which reaffirms that the mechanical allodynia is not correlated with increased weight gain, but is due to the components within our HFD regimen (Supplementary Fig. 1). In addition, fasting glucose levels greater than 250 mg/dL were considered hyperglycemic which no animals ever reached. Overall, we verified that mice placed on a HFD did not develop pain influencing conditions, such as obesity and hyperglycemia, so therefore are not confounding factors that influenced the mechanical allodynia.

As previously mentioned, it is well known that SFAs can cause robust inflammation^7^. There are multiple mechanisms in which SFAs can accomplish this. Important for this study is the ability of SFAs with carbon chain lengths of C13-C18 to activate Toll-like receptors, CD36, and FFA1/GPR40/120^39–41^. The diet used in this study consists of 60% kcal from fat, 92.5% of which is saturated fat. After consuming this HFD male and female mice had a significant increase in the levels of circulating FFAs (Fig. 6). This increase in FFAs would activate upstream receptor pathways that would induce upregulation of protein kinase C (PKC) g. PKCg is activated in IB4+ sensory neurons during hyperalgesic priming and one of the reasons we investigated if a HFD could induce behavioral sensitization, or cause hyperalgesic priming. We are the first to reveal the ability of a HFD to sensitize animals to an innocuous stimulus. Prior to the administering subthreshold PGE_2_, HFD mice had similar mechanical paw withdrawal thresholds to chow animals, which corroborates with previous findings that HFD alone does not induce a pain state ^5,14,42^. After subthreshold PGE_2_, HFD is revealed to be a catalyst that exacerbates pain responses to non-painful stimuli in both males and female mice in the absence of any pathological conditions (Fig. 2). Though this priming effect only became significant in females, males were also observed to develop a notable priming effect at three-hour post-injection albeit with a faster resolution of mechanical allodynia (Fig. 2).

To investigate the state of IB4+ sensory neurons in this model, IHC staining for ATF3 was performed in lumbar DRG (L3-L5). We observed a diet-dependent increase in ATF3 expression in HFD mice in the ipsilateral and contralateral DRGs which reveals that ATF3 upregulation occurs independent of PGE_2_. This corroborates a similar finding that reported an increase in ATF3 expression in DRG neurons after 8 weeks on a specialized PUFA diet^4^. Male mice on our HFD demonstrated a marked statistical increase in ATF3 in IB4+ neurons, while female mice did not show a significant difference, biologically ATF3 expression is zero, so any expression is biologically relevant (Fig. 3). Previous studies that have looked at how diet can exacerbate existing pain states have only done research with male rodents^4,5,17^. This highlights the need to use both sexes while investigating as it has become more apparent recently that males and females do not develop pain using the same mechanisms^43,44^.

We believe the upregulation of circulating SFAs from the diet can contribute to allodynia by sensitizing TRP channels^30^. After 9 weeks on diet, male and female DRGs were cultured for calcium imaging experiments. We measured the percentage of cells that responded to typical application of capsaicin to see if the HFD sensitized the TRPV1 channel on sensory neurons. We saw that both HFD-fed male and female mice had a significant increase in the percent of cells that responded to capsaicin (Fig. 3). We chose to use capsaicin and not PGE_2_ in the calcium imaging experiments because it is well described that PGE_2_ does not induce a robust calcium imaging response, so at the level of the cell, there is no way to differentiate subthreshold from threshold {Sheahan, 2018 #8}.

After our HFD regimen, we did not observe a diet-dependent increase in macrophages nor satellite glial cells (SGCs) in the DRG, suggesting that they may not be the major cell type involved in precipitating diet sensitization. SFAs can bind to TLR4 on macrophages and cause release of pro-inflammatory cytokines^9,47,48^. Palmitic acid (PA) (C16) is the most abundant SFA in Western diet and in circulation^46^. The exact cell type that drives this reaction is unknown, but it could be a combination of macrophages and neurons or potentially one cell type could be more important in different sexes. More studies will have to be done to figure out which cell type is the most important in this model.

In conclusion, this study highlights the impact of HFD on developing a diet-induced hyperalgesic primed state, or diet sensitization, in mice through administering an innocuous stimulus in the absence of any gross conditions or injury. It further illustrates and supports a growing body of literature demonstrating that diet plays an important player in influencing the pain status of an individual and is the first to demonstrate the influential role in which a short exposure to a HFD is a risk factor for developing painful, inflammatory conditions. Health professionals therefore may need to also evaluate the dietary components of patients when developing a treatment and prevention plan preceding the onset of symptomologies associated with obesity and diabetes.

## Materials and methods

### Animals

All animal experiments were conducted in accordance to ARRIVE guidelines and approved and curated protocols by the Institutional Animal Care and Use Committee (IACUC) of the University of Texas at Dallas. We used males and females to determine if they possess different mechanisms that drive the development of diet sensitization. Mice were housed (4–5 per cage) in a temperature-controlled facility kept around 21 °C and 50% humidity and maintained on a 12-h light/dark cycle (lights on from 6:00am to 6:00 pm). Animals had ad-libitum access to their respective diet and water and were 6-weeks old at the beginning of the experiment (males: 19.734 g ± 0.444; females: 15.634 g ± 0.254). Pirt-GCaMP3 mice were a generous gift from Xinzhong Dong (John Hopkins University) while WT C57BL/6J mice (stock no. 000664) were purchased from Jackson Laboratory^49^. Pirt-GCaMP3 mice have a genetically encoded calcium indicator, GCaMP3, expressed under the control of the Pirt promoter, expressed in 95% of sensory neurons^49^. All Pirt-GCaMP3 mice used were heterozygotes for Pirt-GCaMP3. They were bred in-house for behavior, IHC, and calcium imaging experiments.

### Diet

Animals were randomly assigned either control (chow) (laboratory chow diet with 5.3% kcal from fat, 54.7% kcal from carbohydrates, and 18.0% kcal from proteins (LabDiet ProLab RMH 1800)) or high-fat diet (HFD) (adjusted calories diet with 60.3% kcal from fat (92.5% saturated, 2% monounsaturated, 5.5% polyunsaturated), 21.4% kcal from carbohydrates, and 18.3% kcal from proteins (TD.08500, Teklad Custom Diet, Envigo)) with consideration to ensure the weights between the diet groups were no different prior to diet initiation. Littermates were randomly placed on their respective diet at age 6 weeks for 9 weeks. Animals were weighed weekly (Monday) between 8:00am and 1:00 pm for 8 weeks on their respective diets, and diets were replenished weekly after weighing the animals in a controlled environment (HFD males: n = 14; Chow males: n = 14; HFD females: n = 15; Chow females: n = 14).

### Fasting glucose

Fasting glucose levels were performed by 14 h overnight removal of food (starting at 6 pm). Since mice are coprophagic, they were placed in a fresh cage with fresh bedding, a new empty food hopper, and used nest (to control for stress levels) and glucose testing was performed the next morning at 8:00am in a quiet environment. The mice were allowed five minutes to become calm after the lid of their cages were removed. Once mice were calm, the tail was clipped ~ 2 mm, and blood was quickly taken from the tail tip and assessed using an Alpha Trak 2 Veterinary Blood Glucose Monitoring Meter Kit week 0, 4, and 8 (HFD males: n = 7; Chow males: n = 7; HFD females: n = 8; Chow females: n = 8). Mice with fasting glucose levels greater than 250 mg/dL were considered hyperglycemic which no animals used for this experiment reached^38^.

### Serum collection and analysis

Tail blood was collected week 8 or 9 (the day before tissue collection) on diet with 300µL EDTA-lined capillary tubes (Sarstedt, Cat#101093-992) after a ~ 2 mm tail clip was taken which took ~2 min for each mouse. After each capillary tube was filled, they were placed on ice until centrifugation at 15,000 rpm at 4 °C for 15 min to isolate serum. 20µL serum aliquots were stored in 0.6 mL tubes and immediately stored at − 80 °C until analysis. Serum/Fatty Acid Detection Kit (Zenbio, Cat#sfa-1) was used to detect serum NEFA levels (Week 8: HFD males: n = 5; Chow males: n = 3; HFD females: n = 6; Chow females: n = 5) (Week 9: HFD males: n = 5; Chow males: n = 5; HFD females: n = 5; Chow females: n = 5). Serum aliquots were randomized such that the experimenters were blinded to sex and dietary conditions during serum analysis.

### Drugs

PGE_2_ (Cayman Chemical, Cat#14010) was reconstituted in 100% ethanol at a concentration of 1 mg/mL and stored at − 20 °C. On the day of injection, a concentration of 0.005 mg/mL of PGE_2_ was made in sterile 1× phosphate-buffered saline (PBS, pH 7.4) right before injections took place. A stock of 10 mM capsaicin (Sigma-Aldrich, Cat#M2028) was made in 100% ethanol and stored at 4 °C. On the day of calcium imaging capsaicin was diluted to 0.00025 mM in bath solution, described below (see Calcium Imaging).

### Injections

Intraplantar injections were administered to the left hind paw (ipsilateral). A Hamilton syringe (Hamilton, Cat#80501) was used with a disposable 30-gauge ½ inch needle (BD, Cat#305106). Mice were covered with a red towel (to minimize stress) with the left paw lifted, and PGE_2_ was injected at 100 ng/20 µL into the paw pad of the second digit for all animals. After injections, mice were placed in the behavior rack until the time points were collected.

### Behavior

Mice were placed in an elevated rack containing separate compartments with a wire mesh bottom for habituation and behavioral testing. Each compartment was 11.43 cm in length and 5.08 cm in width and was separated by a transparent plexiglass divider. Mice are first acclimated to the behavior room in their own home cage for 30 min, then they were habituated to the behavior rack for 1–2 h for three days before to PGE_2_ administration and habituated for 1–2 h along with measuring baseline sensitivity for 1 and 2 days before PGE_2_ administration). On the day of PGE_2_ administration, the mice were left in the rack to habituate for 1–2 h to become calm. Mechanical sensitivity was assessed using flexible von Frey filaments (Stoelting, Cat#58011) to evaluate the ipsilateral hind paw based on the up-down experimental paradigm^13,51,52^. 2 baseline measurements were taken 1 to 2 days before the day of injections, and again, before PGE_2_ administration or the day of PGE_2_ administration. The average was taken between the 2 baseline measurements for each animal. One animal displayed heightened baseline/naïve pain sensitivity (i.e., < 0.4 g) and was excluded from subsequent analyses. Paw withdrawal threshold was assessed 3 h, 24 h, and 48 h post PGE_2_ injection during week 8 on diet and were done between the hours of 10:00 am and 5:00 pm in a controlled, quiet environment (HFD males: n = 10; Chow males: n = 11; HFD females: n = 11; Chow females: n = 10). Mice were randomly placed into each compartment such that the experimenter was blinded to dietary conditions during behavioral experiments.

### Tissue collection

Mice were anesthetized with isoflurane and then euthanized by decapitation. Lumbar DRGs (L3–L5) were collected on week 9 on diet. If tissues were used for IHC, animals were intracardially perfused with 1x PBS followed by 4% PFA made in 1x PBS. DRGs were then post-fixed in 4% PFA for 4 h and cryoprotected using 30% sucrose (Sigma-Aldrich, Cat#S0389) diluted in 1× PBS in at 4 °C and replaced with 30% sucrose every 24 h until the tissue sank. Once the tissue had sunk, it was embedded in optimal cutting temperature (OCT) (Thermo Fisher Scientific, Cat#50-363-773) compound and stored at either − 20 or − 80 °C until sectioning. DRGs used for calcium imaging were collected fresh. and cultured based on the protocol below.

### Tissue isolation

Cultured primary DRG neurons from thoracic and lumbar (T12–T13, L1–L6) were used for calcium imaging experiments. DRGs were dissected and placed in chilled HBSS solution. The neurons were then digested in collagenase A (1:1; A (Sigma-Aldrich, Cat#10103586001):HBSS (Gibco, Cat#14-170-112)) for 20 min at 37 °C, collagenase D (1:1:10%; D (Sigma-Aldrich, Cat#1188866001):HBSS:papain (Sigma-Aldrich, Cat#10108014001) for 20 min at 37 °C, and then placed in a Trypsin Inhibitor solution (1:1:1; Trypsin (Sigma-Aldrich, Cat#10109886001):BSA: Media) for trituration. Media is DME/F-12 1:1 (1) with 2.50 mM l-Glutamine and 15 mM HEPES buffer (HyClone, Cat#SH30023) supplemented with 10% Fetal Bovine Serum (HyClone, Cat#SH30088.03) and 1% Penicillin Streptomycin (Fisher Scientific, Cat#15070063). After trituration, cells were filtered through a 70 μm cell strainer (Corning, Cat#CLS431751), pelleted, buffer removed, and then resuspended in 200 μL of Media. Plates used were 35 mm Petri dish, 10 mm Microwell, and No. 1.5 cover glass (MatTek Corporation, Cat#P35GC-1.5-C) that were coated in facility with a 2 µg/mL poly-D lysine solution (Sigma-Aldrich, Cat#P0899). Cells were plated in a 200 μL bubble on the center of the plate to rest in an incubator set at 37 °C with 5% CO2 for 2 h before filling the rest of the well with Media. Cells were used the next day for calcium imaging.

### Calcium imaging

DRGs were cultured as described below and imaged the day after plating. Cells were imaged using either GCaMP3 or fura-2 AM (Invitrogen, Cat#F1221). If fura-2 AM was used the dish was loaded with 0.005 mg/mL in loading buffer made in HBSS (Gibco, Cat#14-170-112) containing 0.25% bovine serum albumin (endotoxin free) (Sigma-Aldrich, Cat#A9576) and 2 mM CaCl_2_ for 1 h at 37 °C. Then fura-2 AM was esterified in bath solution (125 mM NaCl (Fisher Scientific, Cat#S271-500), 5 mM KCl (Fisher Scientific, Cat#P217-500), 10 mM HEPES (Sigma-Aldrich, Cat#H4034), 1000 mM CaCl_2_ (Sigma-Aldrich, Cat#21115, 1,000 mM MgCl_2_ (Fisher Scientific, Cat#M35-500), and 2,000 mM glucose (Sigma-Aldrich, Cat#G7528)) at a pH of 7.4 ± 0.05 and mOsm of 300 ± 5 for 30 min at 37 °C. If GCaMP3 was used for imaging, cells were placed in bath and imaged 30 min after being placed in bath. A previous study revealed comparable activity levels between Pirt-GCaMP3 DRG neurons and fura-2 AM stained DRG neurons, so subsequent analyses graphed both data sets together^49^. Capsaicin at 0.00025 mM was applied on cells for 20 s followed by a 120 s wash and then a 10 s application of 50 mM KCl as a positive control. Only neurons were used in analysis which were defined as any cell with a 20% response change from baseline change to 50 mM KCl cells not meeting this requirement were excluded from further analysis. At least 16% change from baseline was counted as a response to 0.00025 mM capsaicin. All experiments were run using the MetaFluor Fluorescence Ratio Imaging Software on an Olympus TH4-100 apparatus. Imaging was done on a 40 × oil objective on the FITC channel. Ratios of bound (excitation wavelength: 340 nm) to unbound (excitation wavelength: 380 nm) fura-2 AM (emission wavelength: 510 nm) were recorded real time and GCaMP3 intensity changes were recorded real time. All imaging data was exported in a Microsoft Excel spreadsheet and analyzed using the algorithms in a Microsoft Excel. Latency to peak was measured by the amount of time it took to reach the peak response to capsaicin and the magnitude response was calculated by the percentage increase from baseline response (prior to capsaicin application) to peak response to capsaicin. Individual cells were excluded if the baseline value was underneath a value of 0.45 or exceeded a value of 1.75 for F_340nm_/F_380nm_ ratio.

### Immunohistochemistry (IHC)

Lumbar (L3-L5) DRGs were cryosectioned at 18 microns and mounted onto a charged slide. Sections were dried on a slide warmer before being stored at − 20 °C. On the day of IHC, slides were dried on a slide warmer for ten minutes and a hydrophobic border was drawn around the sections using a PAP pen (Fisher Scientific, Cat#23-769-533) to ensure the reagents stay on the slide. The slides were subsequently fixed with 4% paraformaldehyde (PFA) (Fisher Scientific, Cat# F79-500) diluted in 1x PBS in the fume hood for five minutes. After three changes of wash buffer (1x PBS containing 0.05% Tween-20 (Sigma-Aldrich, Cat#P1379)), antigen retrieval was performed using three changes of heated 10 mM citrate buffer (2.94% trisodium citrate Sigma-Aldrich, Cat# C8532), 0.05% Tween-20, diluted in ddH_2_O) (pH = 6.0 ± 0.05) for five minutes each. A combination permeabilization/blocking buffer (2% heat-inactivated normal goat serum (Gibco, Cat#16210-072) for IB4+ and ATF-3 antibodies or 5% heat-inactivated normal goat serum for CD68, NeuN, and GFAP antibodies , 1% bovine serum albumin (BSA; VWR, Cat#97061-416), 0.1% Triton X-100 (Sigma-Aldrich, Cat#X100), 0.05% Tween-20, and 0.05% sodium azide (Sigma, Cat#RTC000068) diluted in 1x PBS) was applied for 2 h followed by the appropriate primary antibody cocktail application overnight at 4 °C (Table 1). When applying isolectin GS-IB4, biotin-xx conjugates, CaCl_2_ was added at the same concentration to activate IB4^53,54^. After three changes of wash buffer, the slides were incubated in the appropriate secondary antibodies cocktail for 2 h at room temperature (Table 2). Slides were washed before and after incubating the tissues in 4′,6-diamidino-2-phenylindole (DAPI) for five minutes. Coverslips were mounted onto slides using Gelvatol and cured overnight at room temperature. Sealant was applied around the border of the coverslip to prevent tissue dehydration, and slides were stored at 4 °C until imaging. See Table 1 and 2 for more information on the antibodies used for this study.

**Table 1:** Primary antibodies.

Antibody	Vendor	Raised in	Concentration
Isolectin GS-IB4, biotin-xx conjugates	Invitrogen (I21414)	N/A	1:500
ATF-3	Abcam (ab207434)	Rabbit	1:500
GFAP	Dako (Z0334)	Rabbit	1:500
NeuN	EMD Millipore Corporation (MAB377)	Mouse IgG1	1:500
CD68	Bio-Rad (MCA1957)	Rat IgG2a	1:500
DAPI	Sigma	N/A	1:5000

All primary antibodies were made in the corresponding blocking/permeabilization buffer. CaCl_2_ was added alongside isolectin GS-IB4, biotin-xx conjugates. DAPI was diluted in 1 $\times$ PBS.

**Table 2:** Secondary antibodies.

Antibody	Vendor	Raised in	Concentration
Streptavidin, Alexa Fluor 488	Invitrogen (I21414)	N/A	1:1000
Alexa Fluor 568 goat anti-mouse	Invitrogen (A11004)	Goat	1:1000
Alexa Fluor 647 goat anti-rabbit	Invitrogen (A21245)	Goat	1:1000
Alexa Fluor 568 goat anti-mouse IgG1	Invitrogen (A21124)	Goat	1:1000
Alexa Fluor 488 goat anti-rat	Invitrogen (A11006)	Goat	1:1000

All primary antibodies were made in the corresponding blocking/permeabilization buffer.

### Image acquisition and analysis

Each animal was randomly assigned a letter (A-Z) when sectioning, and images were acquired using a Zeiss Axiobserver 7 Epifluorescent Microscope for DRGs by a blinded experimenter. 2 experimenters analyzed these images and were blinded to sex and dietary conditions throughout the entirety of imaging analysis. ATF3 quantification was presented as the number of ATF3+ cells in the DRG that was calculated through a manual count of the number of ATF3+ cells divided by the area of the DRG. The area of the DRG was measured by drawing a region of interest around the DRG, excluding the axons of the DRG as much as possible through ImageJ Version 2.0.0. In addition, ATF3 quantification was further presented as the percentage of IB4 expressing ATF3 that was calculated as a percentage of the manual count of the number of IB4+ cells expressing ATF3 in the IB4+ cell population in the DRG. (HFD males: 6; Chow males: 4; HFD females: 6; Chow females: 3). CD68 quantification was presented as the number of CD68 + cells in the DRG divided by the total area of DRG. The number of CD68+ cells was quantified through a manual threshold adjustment with ImageJ Version 2.0.0. for each DRG. (HFD males: 6; Chow males: 4; HFD females: 5; Chow females: 3). GFAP quantification was presented as the percentage of neurons surrounded by activated satellite glial cells, GFA+  cells, divided by the total number of neurons, NeuN + cells. GFAP + cells were counted and included in the analysis if it surrounded the neurons. (HFD males: 6; Chow males: 4; HFD females: 5; Chow females: 3). Up to 4 images from each animal were acquired, analyzed, and averaged. There was at least a biological replicate of three animals for each group. All representative images were taken on an Olympus FluoView 3000 RS laser scanning confocal microscope.

### Statistical analysis

All data are represented as mean ± SEM. All graphs and analyses were performed using GraphPad Prism 9.3.1 (GraphPad, San Diego, CA, USA). Weights were analyzed using a two-way ANOVA with Sidak’s post hoc test to observe the influence of diet and sex on the data and compare the differences between each group (HFD males: n = 14; Chow males: n = 14; HFD females: n = 15; Chow females: n = 14). Assessment of fasting glucose levels at each timepoint was analyzed using two-way mixed analysis with Sidak’s post hoc test since fasting glucose was not recorded at every time point for each animal. Behavioral data was analyzed using a two-way ANOVA with Sidak’s post hoc test to observe the influence of time, diet, and sex on the data and the area over the curve (AOC) were analyzed using a two-way ANOVA with Sidak’s post hoc test to analyze the effect of diet and sex. Calcium imaging analysis were analyzed using two-way ANOVA with Sidak’s post hoc test to analyze effect of diet and sex on the trends and form quantitative comparisons between groups. Fisher’s exact test analysis was used to compare the proportions of cells responding to capsaicin between HFD and chow within sex (HFD males: n = 6 mice, 195 cells; Chow males: n = 9 mice, 151 cells; HFD females: n = 8 mice, 239 cells; Chow females: n = 7 mice, 292 cells). For the IHC experiments, each group had at least three animals with up to 4 replicates obtained and averaged for each animal. IHC analysis was then analyzed using three-way ANOVA with Sidak’s post hoc multiple comparison to observe the interactions between diet, sex, and PGE_2_ injection. NEFA fold induction analysis was analyzed using a three-way ANOVA with Sidak’s post hoc multiple comparison to observe the influence of time, diet, and sex on the data and the interactions between each factor. Statistical values for each analysis are also provided in Supplementary Information. All experiments were performed with experimenters blinded to condition. p values *p < 0.05; **p < 0.01; ***p < 0.001; ****p < 0.0001.

## Supplementary Information

Supplementary Information 1.

Supplementary Information 2.

Supplementary Information 3.

## Data Availability

The datasets generated during and/or analyzed during the current study are not included as a supplement or posted publicly because raw data file size was > 240 GB and file types were incompatible to store together. But any data will be available from the corresponding author upon reasonable request.
