# Supplementary material for: High-fat diet causes mechanical allodynia in the absence of injury or diabetic pathology
Source: Sci Rep. 2022 Sep 1;12:14840. doi: 10.1038/s41598-022-18281-x (PMC9437006; doi:10.1038/s41598-022-18281-x)
Supplement: Supplementary file 1 — Supplementary Information 1. [file 41598_2022_18281_MOESM1_ESM.docx]

| Dataset | | Main Effect |  | | Interactions | |  | Multiple Comparisons | | | |
| --- | --- | --- | --- | --- | --- | --- | --- | --- | --- | --- | --- |
|  | | F (DFn, DFd) | *p*-value |  | F (DFn, DFd) | *p*-value |  | Effect | Groups | Week | *p-value* |
| Weight | Males | Diet: F (1, 26) = 0.1692  Time: F (8, 208) = 158.2 | *P=0.6842*  ***p<0.0001*** |  | F (8, 208) = 4.323 | ***p*<0.0001** |  | Diet | Males | 0  1  2  3  4  5  6  7  8 | *p=0.8568*  *p=0.9969*  *p>0.9999*  *p>0.9999*  *p=0.9999*  *p=0.9378*  *p=0.9998*  *p=0.8054*  *p=0.8062* |
|  | Females | Diet: F (1, 27) = 33.91  Time: F (3.306, 89.26) = 216.3 | ***p<0.0001***  ***p<0.0001*** |  | F (8, 216) = 8.931 | ***P<0.0001*** |  | Diet | Females | 0  1  2  3  4  5  6  7  8 | *p=0.9922*  ***p=0.0059***  ***p=0.0004***  ***p<0.0001***  ***p<0.0001***  ***p<0.0001***  ***p<0.0001***  ***p=0.0001***  ***p=0.0007*** |
| Fasting Glucose | Males | Diet: F (1, 29) = 2.992  Time: F (2, 29) = 2.819 | *p=0.0943*  *p=0.0761* |  | F (2, 29) = 0.4002 | p=0.6738 |  | Diet | Males | 0  4  8 | *p=0.4522*  *p=0.5136*  *p=0.9842* |
|  | Females | Diet: F (1, 20) = 4.997  Time: F (1.616, 9.696) = 1.360 | ***p=0.0370***  *p=0.2940* |  | F (2, 12) = 0.2164 | p=0.8085 |  | Diet | Females | 0  4  8 | *p=0.5212*  *p=0.4582*  *p=0.3130* |

Supplementary Table 1. Statistical values for analyses performed within Figure 1. Weights were analyzed using repeated measures Two-Way ANOVA with Sidak’s *post hoc*. Fasting glucose was analyzed using Two-Way mixed analysis ANOVA with Sidak’s *post hoc*. Significance was set at *p*<0.05 for all datasets. Statistically significant values are bolded.

| Dataset | | Main Effect |  | | Interactions | |  | Multiple Comparisons | | | |
| --- | --- | --- | --- | --- | --- | --- | --- | --- | --- | --- | --- |
|  | | F (DFn, DFd) | *p*-value |  | F (DFn, DFd) | *p*-value |  | Effect | Groups | POD | *p-value* |
| Mechanical Sensitivity | Males | Diet: F (1, 19) = 0.6502  Time: F (2.108, 40.05) = 2.419 | *p=0.4300*  *p=0.0993* |  | F (3, 57) = 1.688 | *p=0.1798* |  | Diet | Males | BL  3hr  24hr  48hr | *p=0.9984*  *p=0.4840*  *p=0.9656*  *p=0.9422* |
|  | Females | Diet: F (1, 19) = 8.064  Time: F (3, 57) = 4.894 | ***p=0.0105***  ***p=0.0043*** |  | F (3, 57) = 1.627 | *p=0.1932* |  | Diet | Females | BL  3hr  24hr  48hr | *p=0.9942*  ***p=0.0088***  *p=0.2012*  *p=0.5546* |
| Mechanical Sensitivity  (AOC) | 3hr AOC | Diet: F (1, 38) = 13.22  Sex: F (1, 38) = 1.925 | ***p=0.0008***  *p=0.1734* |  | F (1, 38) = 0.1491 | *p=0.7016* |  | Diet | Males  Females |  | *p=0.0535*  ***p=0.0142*** |
|  | 24hr AOC | Diet: F (1, 38) = 6.022  Sex: F (1, 38) = 3.793 | ***p=0.0188***  *p=0.0589* |  | F (1, 38) = 2.470 | *p=0.1243* |  | Diet | Males  Females |  | *p=0.7851*  ***p=0.0141*** |
|  | 48hr AOC | Diet: F (1, 38) = 1.963  Sex: F (1, 38) = 8.256 | *p=0.1694*  ***p=0.0066*** |  | F (1, 38) = 0.3879 | *p=0.5371* |  | Diet | Males  Females |  | *p=0.8281*  *p=0.2954* |
|  | CumulativeAOC | Diet: F (1, 38) = 9.939  Sex: F (1, 38) = 7.706 | ***p=0.0032***  ***p=0.0085*** |  | F (1, 38) = 1.446 | *p=0.2366* |  | Diet | Males  Females |  | *p=0.3210*  ***p=0.0077*** |

Supplementary Table 2. Statistical values for analyses performed within Figure 2. Mechanical sensitivity was analyzed using a repeated measures Two-Way ANOVA with Sidak’s *post* hoc and effect sizes was analyzed with an Ordinary Two-Way ANOVA with Sidak’s *post hoc*. Statistical significance was set at p<0.05. Statistically significant values are bolded.

| Dataset | | Main Effect |  | | Interactions | |  | Multiple Comparisons | | | |
| --- | --- | --- | --- | --- | --- | --- | --- | --- | --- | --- | --- |
|  | | F (DFn, DFd) | *p*-value |  | F (DFn, DFd) | *p*-value |  | Effect | Groups | POD | *p-value* |
| Calcium Imaging | Fold Change | Diet: F (1, 16) = 71.58  Sex: F (1, 16) = 1.398 | ***p<0.0001***  *p=0.2544* |  | F (1, 16) = 0.2244 | p=0.6421 |  | Diet | Males  Females |  | ***p<0.0001***  ***p<0.0001*** |
|  | Magnitude Response | Diet: F (1, 306) = 8.166  Sex: F (1, 306) = 0.3274 | ***p=0.0046***  *p=0.5676* |  | F (1, 306) = 0.06150 | p=0.8043 |  | Diet | Males  Females |  | *p=0.1505*  ***p=0.0434*** |
|  | Latency to Peak | Diet: F (1, 291) = 0.002815  Sex: F (1, 291) = 25.26 | *p=0.9577*  ***p<0.0001*** |  | F (1, 291) = 0.4161 | p=0.5194 |  | Diet | Males  Females |  | *p=0.8640*  *p=0.8891* |

Supplementary Table 3. Statistical values for analyses performed within Figure 3. Fold change, magnitude response, and latency to peak were analyzed with an Ordinary Two-Way ANOVA with Sidak’s *post hoc*. Statistical significance was set at *p*<0.05. Statistically significant values are bolded.

| Dataset | | Main Effect |  | | Interactions | |  | Multiple Comparisons | | |
| --- | --- | --- | --- | --- | --- | --- | --- | --- | --- | --- |
|  | | F (DFn, DFd) | *p*-value |  | F (DFn, DFd) | *p*-value |  | Effect | Groups | *p-value* |
| ATF3 Analysis | ATF3+ Neurons | Diet: F (1, 30) = 7.620  Sex: F (1, 30) = 1.296  PGE_2_ Injection: F (1, 30) = 2.564 | ***p=0.0098***  *p=0.2639*  *p=0.1198* |  | F (1, 30) = 0.6555 | *p=0.4245* |  | Diet  PGE_2_ Injection | Males: Ipsilateral Male HFD vs Ipsilateral Male Chow  Females: Ipsilateral Female HFD vs Ipsilateral Female Chow  Males: Contralateral Male HFD vs Contralateral Male Chow  Females: Contralateral Female HFD vs Contralateral Female Chow  Males: Ipsilateral Male HFD vs Contralateral Male HFD  Females: Ipsilateral Female HFD vs Contralateral Female HFD  Males: Ipsilateral Male Chow vs Contralateral Male Chow  Females: Ipsilateral Female Chow vs Contralateral Female Chow | ***p=0.0421***  *p=0.9960*  *p=0.9871*  *p>0.9999*  *p=0.1788*  *p=0.9994*  *p>0.9999*  *p>0.9999* |
|  | IB4+ Neurons | Diet: F (1, 30) = 3.588  Sex: F (1, 30) = 2.316  PGE_2_ Injection: F (1, 30) = 1.439 | *p=0.0679*  *p=0.1386*  *p=0.2397* |  | F (1, 30) = 1.526 | *p=0.2263* |  |  |  |  |

Supplementary Table 4. Statistical values for analyses performed within Figure 4. ATF3 analysis were analyzed using Ordinary Two-Way ANOVA with Sidak’s *post hoc*. Significance was set at *p*<0.05 for all datasets. Statistically significant values are bolded.

| Dataset | | Main Effect |  | | Interactions | |  | Multiple Comparisons | | | |
| --- | --- | --- | --- | --- | --- | --- | --- | --- | --- | --- | --- |
|  | | F (DFn, DFd) | *p*-value |  | F (DFn, DFd) | *p*-value |  | Effect | Groups | Week | *p-value* |
| CD68 Analysis | Number of CD68+ Cells | Diet: F (1, 25) = 1.344  Sex: F (1, 25) = 3.698  PGE_2_ Injection: F (1, 25) = 0.8066 | *p=0.2572*  *p=0.0659*  *p=0.3777* |  | F (1, 25) = 0.6846 | *p=0.4158* |  |  |  |  |  |
| GFAP Analysis | Number of GFAP+ Cells | Diet: F (1, 26) = 0.4788  Sex: F (1, 26) = 1.370  PGE_2_ Injection: F (1, 26) = 3.162 | *p=0.4951*  *p=0.2524*  *p=0.0871* |  | F (1, 26) = 1.864 | *p=0.1839* |  |  |  |  |  |
|  |  |  |  |  |  |  |  |  |  |  |  |

Supplementary Table 5. Statistical values for analyses performed within Figure 5. CD68 and GFAP analysis were analyzed using Ordinary Two-Way ANOVA with Sidak’s *post hoc*. Significance was set at *p*<0.05 for all datasets. Statistically significant values are bolded.

| Dataset | | Main Effect |  | | Interactions | |  | Multiple Comparisons | | |
| --- | --- | --- | --- | --- | --- | --- | --- | --- | --- | --- |
|  | | F (DFn, DFd) | *p*-value |  | F (DFn, DFd) | *p*-value |  | Effect | Groups | *p-value* |
| NEFA Analysis | Week 8  Fold Change | Diet: F (1, 15) = 44.13  Sex: F (1, 15) = 1.452 | **p<0.0001**  p=0.2468 |  | F (1, 15) = 0.02746 | *p=0.8706* |  | HFD | Males  Females | ***p*=0.0010**  ***p*=0.0003** |
|  | Week 9  Fold Change | Diet: F (1, 16) = 143.2  Sex: F (1, 16) = 1.106 | **p<0.0001**  p=0.3086 |  | F (1, 16) = 0.7482 | *p=0.3998* |  | HFD | Males  Females | ***p<0.0001***  ***p<0.0001*** |

Supplementary Table 6. Statistical values for analyses performed within Figure 6. NEFA analysis was analyzed using Ordinary Two-Way ANOVA with Sidak’s *post hoc*. Significance was set at *p*<0.05 for all datasets. Statistically significant values are bolded.
