# Supplementary material for: High-fat diet causes mechanical allodynia in the absence of injury or diabetic pathology
Source: Sci Rep. 2022 Sep 1;12:14840. doi: 10.1038/s41598-022-18281-x (PMC9437006; doi:10.1038/s41598-022-18281-x)
Supplement: Supplementary file 2 — Supplementary Information 2. [file 41598_2022_18281_MOESM2_ESM.docx]

**Supplementary Figure 1.** Diet sensitization cumulative effect is not correlated with weight in either sex. (a, b) Correlation analyses comparing weights at week 8 and the cumulative area over the curve revealed no significant correlation between the two variables in either sex.
