# Supplementary figures and images for: High-fat diet causes mechanical allodynia in the absence of injury or diabetic pathology
Source: Sci Rep. 2022 Sep 1;12:14840. doi: 10.1038/s41598-022-18281-x (PMC9437006; doi:10.1038/s41598-022-18281-x)

**a**

### Weights v. Diet Sensitization AOC

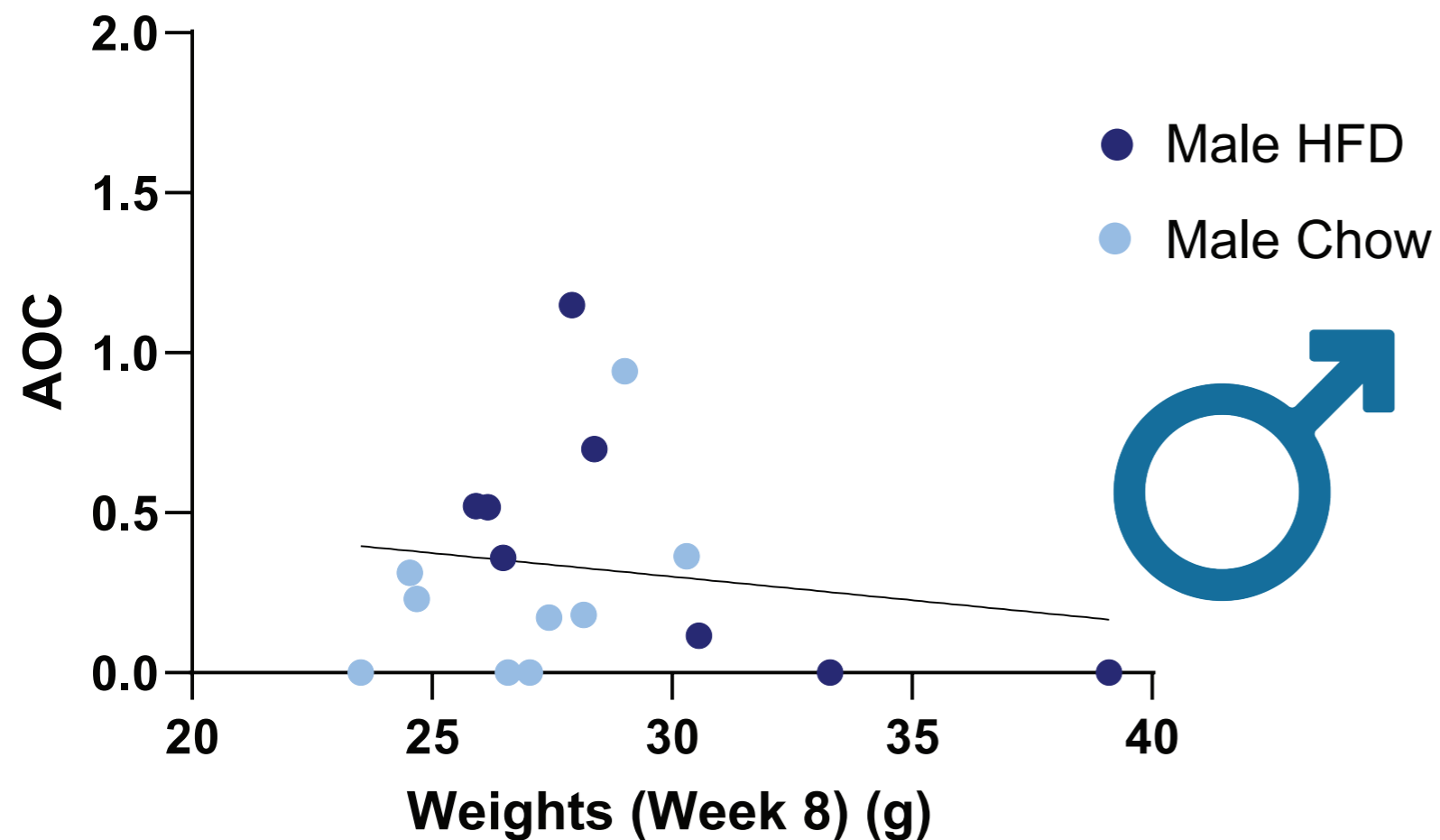**b**

### Weights v. Diet Sensitization AOC

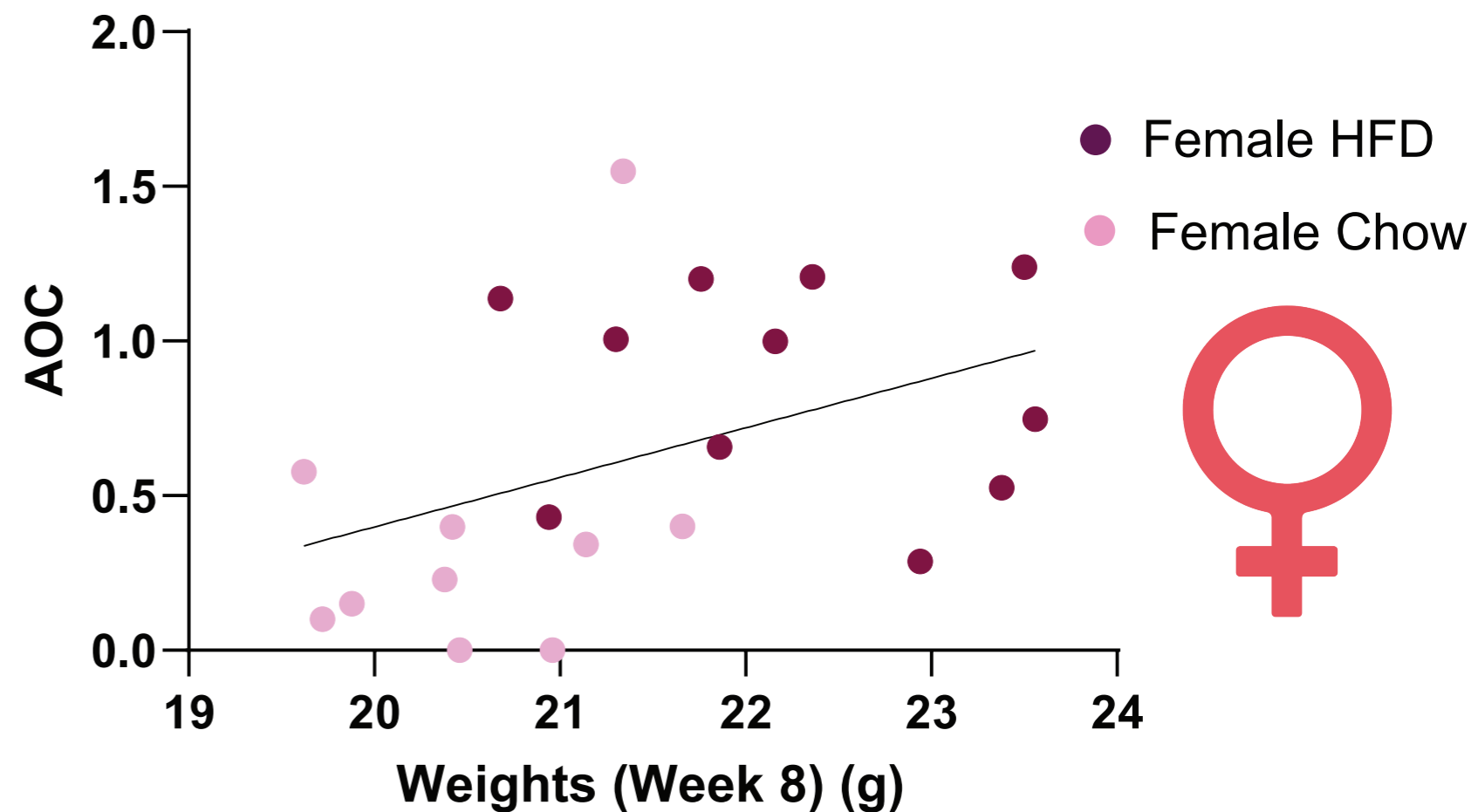

Supplement: Supplementary file 3 — Supplementary Information 3. [file 41598_2022_18281_MOESM3_ESM.pdf]
